# Modulation of PPAR Expression and Activity in Response to Polyphenolic Compounds in High Fat Diets

**DOI:** 10.3390/ijms17071002

**Published:** 2016-06-29

**Authors:** J. Abraham Domínguez-Avila, Gustavo A. González-Aguilar, Emilio Alvarez-Parrilla, Laura A. de la Rosa

**Affiliations:** 1 Coordinación de Tecnología de Alimentos de Origen Vegetal, Centro de Investigación en Alimentación y Desarrollo A. C., Carretera a La Victoria km 0.6, AP 1735, CP 83304 Hermosillo, Sonora, Mexico; abrahamdominguez9@yahoo.com (J.A.D.-A.); gustavo@ciad.mx (G.A.G.-A.); 2 Departamento de Ciencias Químico-Biológicas, Instituto de Ciencias Biomédicas, Universidad Autónoma de Ciudad Juárez, Anillo Envolvente del PRONAF y Estocolmo s/n, CP 32310 Ciudad Juárez, Chihuahua, Mexico; ealvarez@uacj.mx

**Keywords:** PPAR, polyphenols, high fat, gene expression

## Abstract

Peroxisome proliferator-activated receptors (PPAR) are transcription factors that modulate energy metabolism in liver, adipose tissue and muscle. High fat diets (HFD) can negatively impact PPAR expression or activity, favoring obesity, dyslipidemia, insulin resistance and other conditions. However, polyphenols (PP) found in vegetable foodstuffs are capable of positively modulating this pathway. We therefore focused this review on the possible effects that PP can have on PPAR when administered together with HFD. We found that PP from diverse sources, such as coffee, olives, rice, berries and others, are capable of inducing the expression of genes involved in a decrease of adipose mass, liver and serum lipids and lipid biosynthesis in animal and cell models of HFD. Since cells or gut bacteria can transform PP into different metabolites, it is possible that a synergistic or antagonistic effect ultimately occurs. PP molecules from vegetable sources are an interesting option to maintain or return to a state of energy homeostasis, possibly due to an adequate PPAR expression and activity.

## 1. Introduction

Peroxisome proliferator-activated receptors (PPAR) are classified as part of the nuclear hormone receptor superfamily, whose endogenous ligands include fatty acids and eicosanoids; once activated, they will heterodimerize with the retinoid X receptor (RXR) and bind to specific DNA sequences (PPAR response elements (PPRE)) that will ultimately lead to the transcription of genes related to maintaining adequate lipid and glucose metabolism [1]. Three members are currently known as: α, β/δ and γ. PPARα is expressed in liver, heart, small intestine, skeletal muscle and brown adipose tissue (BAT); its natural ligands include polyunsaturated long chain fatty acids (such as linoleic acid, docosahexaenoic acid and eicosapentaenoic acid), oxidized fatty acids, arachidonic acid derivatives like leukotriene B4, and others, such as endocannabinoids; its physiologic role is mostly related to fatty acid metabolism [2,3,4,5]. PPARβ/δ is ubiquitously expressed (high expression has been reported in skin, skeletal muscle, adipose tissue, heart and others); its endogenous ligands include fatty acids, triacylglycerols (TAG), prostacyclin and retinoic acid; its physiologic roles include improving insulin sensitivity and fatty acid oxidation in adipose tissue, favoring lipid over glucose oxidation in skeletal muscle and others [6]. Two PPARγ proteins derived from different promoters and 5′ exons are currently known as PPARγ1 (expressed ubiquitously) and PPARγ2, which is expressed mainly in adipose tissue, where it is responsible for adipocyte differentiation and fat storage that prevents lipotoxicity and allows for optimum insulin signaling; its endogenous ligands include polyunsaturated fatty acids and endocannabinoids [5,7,8,9]. Diet components can either downregulate or upregulate PPAR expression and activity. Diet-induced PPAR downregulation can be considered as a negative effect, since it is often present alongside conditions like dyslipidemia [10], hypoadiponectinemia [11], insulin resistance/diabetes [12], chronic inflammation [13], lipotoxicity [14], diabetic nephropathy [15], diabetic retinopathy [16] and several others; such downregulation can be seen when routinely consuming Western-type diets, which are rich in fat. PPAR upregulation can be considered a positive effect that can occur by favoring vegetable consumption over Western-type diets; this will stimulate β-oxidation, insulin sensitivity, adiponectin secretion and glucose uptake and catabolism, all of which can be impaired in diabetic, obese or dyslipidemic patients. Furthermore, pharmaceutical PPAR agonists have been developed to act specifically on PPARα (fibrates) or PPARγ (thiazolidinediones or glitazones); they are currently on the market for diabetic patients with the purpose of inducing PPAR activity in peripheral cells. However, pharmacologic PPAR activation can have negative side effects, such as increased food intake, increased cardiac and hepatic adipogenesis and others [7]. Some compounds of vegetable origin, such as polyphenols (PP), can also exert similar activity as the synthetic PPAR agonists with minimal side effects and are thus of key interest. The name PP implies phenolic moieties as a defining characteristic, but they can also be subclassified according to other distinguishing features [17]. PP are present in fruits, vegetables, medicinal plants, folk remedies, in aqueous or organic extracts obtained from these sources or individually in solution, tablet, etcetera. The literature was reviewed for original articles, highlighting the effects of PP from diverse sources, when administered as part of a high fat diet (HFD), on PPAR expression or activity. PPAR modulation was related to changes in weight, serum lipid profile, adiposity, hepatic steatosis and modulation of other enzymes or transcription factors related to lipid homeostasis in the liver, muscle and adipose; therefore, all of these effects were summarized, as well. The model used, dosage, length of study and PP source and composition were also included (if no PP composition were reported by the original authors, another source was cited to best approximate the probable composition). Most studies reported an upregulation of PPAR mRNA, protein expression or activity, along with an increase in serum or hepatic lipid clearance that ameliorated the harmful effects of an HFD. Therefore, PP act as regulators of the genetic expression of the PPAR family that favor health.

## 2. Effect of Polyphenolic Compounds from Different Sources on PPAR Expression or Activity

Articles are listed according to PP source (fruit juices, other beverages, berries, other edible plants or fruits, edible crops, medicinal plants or folk remedies and pure PP). Effects on PPAR expression or activity are mostly reported in adipose tissue, muscle and liver. Table 1 summarizes the major findings of the discussed papers.

### 2.1. Effect of PP Derived from Fruit Juices

*Moro* oranges are cultivated in the Mediterranean region and are also referred to as blood oranges, due to their intense red coloration that indicates high anthocyanin concentration. Chemical characterization indicates that cyanidin 3-glucoside and cyanidin 3-(6″-malonylglucoside) are the main compounds found in *Moro* orange juice [18]. Salamone et al. [19] administered an HFD (60% calories from fat, 5.2 kcal/g) to male C57BL6/J mice while substituting water with *Moro* orange juice in the treatment group during a 12-week period. *Moro* orange juice hindered weight gain, enhanced insulin sensitivity (as determined by an insulin tolerance test), decreased TAG (triacylglycerols), TC (total cholesterol), hepatic steatosis and hepatic TAG content. A hepatic PPARα mRNA decrease was reported in the HFD group and an increase in the *Moro* orange juice group. These findings indicate that *Moro* orange juice exerted a corrective effect on serum and hepatic lipids by activating the PPARα pathway that favored lipid oxidation and hindering lipid accumulation.

Noni juice is extracted from the fruit of the *Morinda citrifolia* L. tree, which is native to Southeast Asia and Australia. Lin et al. [20] fed male golden Syrian hamsters a low fat diet (7% fat/0% cholesterol) or an HFD (12% fat/0.2% cholesterol) to induce hyperlipidemia during one week; afterwards, animals from both groups received either distilled water or noni juice (3, 6 or 9 mL/kg BW) for the following six weeks. Results showed no difference in BW (body weight) or weight increase between groups; a reduction in heart, liver and visceral fat in the noni groups fed HFD was observed. Noni juice decreased serum TC and TAG, while also increasing HDL (high density lipoprotein); hepatic TC and TAG were also reduced to levels similar to the control (particularly at the highest dose). Hepatic PPARα mRNA expression was decreased in the HFD group, which was prevented by the noni juice treatments. SREBP2 (sterol regulatory element-binding protein 2) and HMG CoA (hydroxymethylglutaryl coenzyme A) reductase expression was similar in all groups, indicating no effect at the transcriptional level of these genes, which are related to cholesterol metabolism. Furthermore, FAS (fatty acid synthase) expression was unaffected, while SREBP1c expression was increased by the HFD, but was maintained similar to the control group by the two highest doses of noni juice, indicating a partial inhibitory effect on fatty acid synthesis. PP composition of noni juice reported by the authors was (in decreasing concentration): phenolic acids (gentisic, *p*-hydroxybenzoic, chlorogenic, caffeic, *p*-anisic, ferulic and gallic acid) and flavonoids (hesperidin, naringin and epicatechin). A synergistic effect of these PP was likely responsible for the described changes.

### 2.2. Effect of PP Derived from Other Beverages

Green tea is a very popular beverage in many countries, partly due to its numerous health effects. Tian et al. [21] extracted green tea PP and administered them in increasing concentrations (0.8, 1.6, 3.2 g/L) in the drinking water of 30 male Wistar rats that were fed HFD for a 26-week period. Glycemia increased in all groups with respect to the control, but the PP treatments presented lower values than the untreated HF (high fat) group. Serum insulin increased in all groups. TC and TAG increased in all groups, but were similar to the control group in the 1.6 and 3.2 g/L group, respectively. Adiponectin mRNA expression in adipose tissue decreased in the HF group, while the 1.6 and 3.2 g/L groups prevented such a decrease, maintaining mRNA expression similar to the control. Serum adiponectin concentration in the HF group decreased; treatments maintained a similar adiponectin concentration to the control group. This pattern was repeated once again in PPARγ mRNA and protein concentration in adipose tissue, while its phosphorylation was increased in the HF group and maintained similar to the control by all PP treatments. PPARγ phosphorylation can enhance or decrease activity, depending on the phosphorylating enzyme and specific residue [5]; in this case, phosphorylation serves as an inhibiting modification. Decreased PPARγ phosphorylation and increased PPARγ expression induced by PP treatments was responsible for preventing hypoadiponectinemia, thereby allowing adequate adiponectin signaling (increased β-oxidation, insulin sensitivity and glucose uptake; decreased gluconeogenesis). The authors also highlight that PPARγ transcription is dependent on several factors, such as phosphorylation of it and its receptors. Reported green tea PP consist mainly of catechins: (−) epicatechin, (−) epicatechin-3-gallate, (−) epigallocatechin, (−) epigallocatechin-3-gallate, (+) catechin and (+) gallocatechin, of which (−) epigallocatechin-3-gallate is one of the most studied and is considered the most bioactive [22]. Other authors have also shown interest in the effect of green tea catechins: Murase et al. [23] fed C57BL/6 mice a control low fat diet (5% TAG) or an HFD (30% TAG) with or without 0.5% green tea catechins extracted and concentrated from green tea (reported as epigallocatechin gallate, epigallocatechin, epicatechin gallate, epicatechin, gallocatechin, gallocatechin gallate and others), while also making the mice swim (as a form of exercise) for 30 min three times per week for 15 weeks. The HF group increased in BW and visceral fat weight, which was reduced in all treatment groups; also, catechins’ effect was enhanced by exercise. Treatments decreased serum TAG and non-esterified fatty acid concentration to values lower than the control group. Muscular and hepatic β-oxidation was increased by catechins and exercise. Results suggest an effect on PPAR, so the authors sought to determine if green tea catechins were acting as PPARα/δ ligands, but found no such results, thus proposing a secondary (unknown) mechanism by which catechins could be acting. Contrasting results of Tian et al. and Murase et al. may be due to differences in model animal (rat versus mouse), the tissue studied (adipose versus liver and muscle), PPAR isoform (γ versus α and δ) and methodology (real-time PCR versus reporter gene). However, another often overlooked difference is the presence of other compounds that may act synergistically or antagonistically with the molecule of interest, exemplified by an article published by Chen et al. [24], where green tea, black tea or isolated epigallocatechin gallate was administered to rats fed HFD (15% fat) for six months. Green and black tea increased hepatic expression of PPARα mRNA (among others), but the catechin by itself had no effect, yet it was able to increase thermogenesis (via an increase in PPARγ and uncoupling protein 2 (UCP2) gene expression) in adipose tissue.

Together with green tea, coffee is also a popular PP-rich beverage consumed around the world. Murase et al. [25] fed male C57BL/6 mice a control diet (5% *w*/*w* fat), an HFD (30% *w*/*w* fat) or an HFD with coffee PP extract (HFD + 0.5 or 1.0% *w*/*w* coffee PP) for 15 weeks. PP treatment suppressed weight gain and WAT (white adipose tissue) mass (relative to the HFD group) without any differences in energy intake. HFD increased liver weight and liver lipid accumulation, which the PP treatment was able to abolish. Hepatic mRNA expression (determined at the second week of feeding) revealed a downregulation in lipogenic genes, while PPARα/γ expression remained unaffected by the PP treatments. PP had no effect on PPARγ (in WAT) or PPARδ (in BAT and skeletal muscle) mRNA expression. Luciferase assays confirmed a null effect on PPAR; in a luciferase assay, the regulatory region of the gene of interest is cloned in a vector that will express the enzyme luciferase in response to a stimulus, thereby revealing if the stimulus is capable of inducing gene expression. According to these results, coffee PP exert their effects thorough a PPAR-independent mechanism. Coffee PP extract contained 5-caffeoylquinic acid, 3,5-dicaffeoylquinic acid, 5-feruloylquinic acid and isomers of these; no caffeine was present.

Dried leaves of *Ilex paraguariensis*, also known as yerba maté, are commonly used in South America to prepare an infusion (maté); its biological properties have been increasingly studied as consumption reaches other regions of the world [26]. Arçari et al. [27] fed male Swiss (Sw/Uni) mice an HFD for eight weeks to induce obesity; animals were then treated with a daily dose of aqueous extract of roasted yerba maté (1.0 mg/kg BW) or water for another eight weeks. A control group of mice was fed a standard diet for the previously described 16 weeks. Results showed a decrease in BW by yerba maté treatment without affecting food consumption; serum TC, LDL (low density lipoprotein), TAG and glycemia also decreased. PPARγ (in WAT and BAT) and UCP1 (in BAT) mRNA expression decreased in the HFD group; yerba maté maintained expression levels similar to the control, which indicates an anti-obesity effect. Yerba maté extract contained 5-caffeoylquinic acid and caffeic acid as the main PP; caffeine and theobromine were also present. Caffeine and theobromine are methylxanthine alkaloids typically known as β-adrenergic receptor agonists; their contribution to the effects listed may not be due to a direct action on PPAR, but from the stimulation of thermogenic proteins (UCP) via β-adrenergic receptors. In humans [28] and in a mice model [29], a combination of a PPAR agonist and a β-adrenergic receptor agonist can impact adipose mass and serum lipids by a crosstalk of both pathways that increases adipose tissue’s thermogenic ability. In spite of their effects on lipid metabolism, the presence of caffeine or theobromine can be undesired when adrenergic simulation is not well tolerated, thereby limiting the generalized use of yerba maté or other caffeine-containing drinks; in such cases, the effects of PP alone are preferred [30].

### 2.3. Effect of PP Derived from Berries

Blueberries and berries in general owe their attractive coloration to PP pigments that can also induce a biological response. Berries processed into a diverse range of goods leave behind byproducts normally considered waste, but that still retain important amounts of bioactive compounds. These compounds are not fully used for other purposes and represent a significant percentage of the edible portion. Kim et al. [31] fed male Syrian golden hamsters a control diet or an HFD (37% calories from fat and 0.15% cholesterol) enriched with either 8% blueberry pomace, 6% blueberry pomace ethanol extract or 2% residue from the blueberry ethanol extract for a three-week period. Blueberry treatments decreased plasma VLDL (very low density lipoprotein) and TC. Hepatic PPARα mRNA expression was upregulated by the ethanol extract treatment and downregulated by the remaining treatments as compared to the untreated group; however, ACOX (acyl coenzyme A oxidase) expression was not upregulated by ethanol extract treatment, even though it is a PPARα target. The authors suggest an mRNA increase without effects on downstream genes may indicate that PPARα was not activated. The main class of PP present in blueberries is anthocyanins; delphinidin, cyanidin, peonidin and malvidin (as well as their glycosides) are the most abundant or important ones [32].

Mulberries (*Morus indica* L.) are edible plants whose leaves and fruits have high antioxidant capacity [33]. Ou et al. used mulberry water extracts prepared from mulberry fruits to treat HepG2 cells together with oleic acid as a model of human liver subjected to an HFD [34]. Cells were incubated with 0.5 mM oleic acid (HF) or HF and mulberry water extracts (1, 2 or 3 mg/mL) for 24 h. Intracellular lipid accumulation occurred, but was ameliorated by the 2 and 3 mg/mL treatments. SREBP2 and HMG CoA reductase protein levels were reduced by the 2 and 3 mg/mL treatments, indicating a suppression in cholesterol synthesis. The GPAT (glycerol-3-phosphate acyltransferase) protein level decreased, while the PPARα and CPT1 (carnitine palmitoyltransferase) protein level increased, indicating a suppression in TAG synthesis. Taken together, these results suggest that an in vitro model of hepatosteatosis can be alleviated by hydrophilic molecules present in mulberries, via regulation of the protein concentration of key enzymes involved in cholesterol synthesis, TAG synthesis and fatty acid oxidation. The PP composition of the extract was 5.07% phenolic acids, 8.33% flavonoids and 5.66% anthocyanins; the rest consisted of protein, lipids and polysaccharides. Specifically, the 15 PP molecules detected were (in decreasing amounts): rutin, protocatechuic acid, epigallocatechin gallate, epicatechin, caffeic acid, hydroxyflavin, catechin, naringenin, quercetin, *p*-coumaric acid, resveratrol, hesperetin, gallic acid, ferulic acid and gossypin. In a similar study, Kobayashi et al. [35] used mulberry leaf extract from a related species (*Morus alba* L.) to determine its effect on male Wistar rats fed an HFD (14% beef fat). Animals were divided into a control group (low fat diet), HF group (non-treated) and three treatment groups (250, 500 or 1000 mg/kg·BW·d), where the mulberry leaf extract was given orally six times per week for seven weeks. BW and liver weight were unchanged in all groups; liver cholesterol and TAG increased in all groups relative to the control; plasma TAG and non-esterified fatty acids were lower in the treatment groups. DNA microarray analysis suggested that the hepatic PPARα/δ pathway was upregulated, which induced generalized fatty acid oxidation via the stimulation of α-, β- and ω-oxidation-related genes, while simultaneously downregulating lipogenic gene expression. Quercetin and kaempferol were the main components in the extract. Interestingly, when comparing PP content reported by Ou et al. in *Morus indica* fruit against the *Morus alba* leaf powder analyzed by Kobayashi et al., it is evident that quercetin is the main compound present in the leaf, but not in the fruit, while kaempferol was not reported in the fruit, thereby highlighting that PP composition will almost certainly vary between species and different parts of the plant, which will elicit an effect by a different mechanism.

### 2.4. Effect of PP Derived from Other Edible Plants or Fruits

Grapes are extremely bioactive due to their high anthocyanin content. Table grape powder (5% *w*/*w*), an extractable PP-rich fraction, a non-extractable PP-poor fraction or the combination of both fractions were used to supplement an HFD (44% energy from fat) fed to C57BL/6J mice for a 16-week period. Mice fed the PP-rich fraction and the combination of both fractions decreased their total fat and WAT percentages; mice fed the combination of both fractions had lower liver weights and liver TAG. Liver PPARγ mRNA expression was increased in the untreated HFD group, but reduced by the extractable PP-rich fraction treatment. Reduced insulin resistance and inflammation was also documented [36]. Analysis (HPLC) revealed various glycosides of delphinidin, cyanidin, petunidin, peonidin and malvidin in the PP-rich extract.

Lemon verbena (*Lippia citriodora*, also known as lemon beebrush) is an edible plant cultivated in the United States, South America, Spain, India and China, whose leaves are used for culinary (food seasoning and beverage flavoring) and sometimes medicinal (stomach ache and indigestion) purposes; one of the main PP compounds present is verbascoside (also known as acteoside) [37]. Herranz-López et al. [38] treated 3T3-L1 high glucose-induced hypertrophic adipocytes with *L. citriodora* extract or verbascoside for 48 h. PPARα mRNA expression was induced by both treatments; luciferase assays showed that PPARγ was also being stimulated in a dose-dependent manner. The same group also fed LDL receptor-deficient C57BL/6J mice (LDLr^−^/^−^) an HFD (20% fat, 0.25% cholesterol *w*/*w*), while also administering *L. citriodora* extract dissolved in their drinking water for 14 weeks. *L. citriodora* treatment decreased epididymal and inguinal WAT, TC, TAG and liver lipids; food intake, BAT and insulin sensitivity remained unaffected. PPAR was not assayed in the animal model.

Olives (*Olea europaea* L.) are considered a representative element in Mediterranean diets, either as such or processed into olive oil; a significant literature documents their beneficial effects on human health [39]. Kim et al. [40] fed male Sprague-Dawley rats a control diet (10% fat), an HFD (5.5% oil, 1% cholic acid and 2% cholesterol) and an HFD and olive fruit pulp ethyl acetate extract (100 or 300 mg/kg BW, by oral gavage) for 28 days. Treatments suppressed an increase in serum TC and LDL, but without returning their values to control levels. High-dose treatment prevented a decrease in HDL, and an increase in liver lipids as compared to the untreated HFD group. Serum TAG was unaffected by all treatments. Hepatic HMG CoA reductase protein decreased in all HFD groups, without an apparent effect from the olive fruit pulp extract. ACAT (acetyl-coenzyme A acetyltransferase), CYP7A1 (cholesterol 7α hydroxylase) and PPARα protein expression increased in the HFD group; olive fruit pulp extract suppressed CYP7A1 protein expression and increased PPARα protein expression in a dose-dependent manner. Treatment with olive fruit pulp extract is capable of exerting antiatherosclerotic and cardioprotective actions through PP, mainly hydroxytyrosol, which is present in olives and has been extensively studied. In addition to hydroxytyrosol (detected in the extract), other PP have been reported in olives: oleuropein, tyrosol, 4-hydroxyphenyl acetic acid, protocatechuic acid, caffeic acid and *p*-coumaric acid [41].

Litchi (*Litchi chinensis* Sonn.) is a tropical fruit native to China, whose nutritional properties have only recently been studied as it is commercialized around the world [42]. Yang et al. [43] fed male Syrian golden hamsters either a control diet (low in fat and cholesterol), an HFD (11% *w*/*w* fat and 0.2% *w*/*w* cholesterol) with regular drinking water, HFD and 2.5% *w*/*v* litchi flower water extract or HFD and 5% litchi flower water extract for a six-week period. BW and food consumption did not vary among groups; however, all HFD groups drank more water than the control. Larger visceral adipose tissue was found in the animals fed HFD, except with the 2.5% litchi water extract treatment. Serum TC and TAG increased in all HFD groups, but treatment increases were significantly less than non-treated HFD group. Hepatic LDL receptor mRNA expression decreased in the HFD group; HMG CoA reductase did not change; and CYP7A1 decreased in all HFD groups. Hepatic mRNA expression of FAS trended to a decrease in all HFD groups; hepatic mRNA expression of PPARα decreased in the non-treated HFD group; both treatments presented significantly higher expression than control and HFD groups. In general, these results suggest a minimal effect on the expression of cholesterol metabolism-related genes, accompanied by an important effect on TAG metabolism-related genes that favors β-oxidation and inhibits fatty acid and TAG synthesis. The authors reported flavonoids, condensed tannins, anthocyanins and proanthocyanidins (without specifying individual molecules) in the extract.

*Corchorus olitorius* L. (also called nalta jute or molokheiya) is an edible plant that has been studied because of its high PP content. Wang et al. [44] fed three groups of 15 LDLr^−^/^−^ mice an HFD (15 g cocoa butter, 3 g cholesterol, 82 g of CE-2 diet) with 0% (control group), 1 or 3% *w*/*w* dried *C. olitorius* leaf, powder for an eight-week period. *C. olitorius* leaf powder significantly decreased BW gain, liver weight and liver TAG content; epididymal adipose tissue weight was decreased in the 3% group. The 3% (but not 1%) treatment increased hepatic PPARα mRNA expression, suggesting that PPARα may also require other transcription factors (PPARγ?) to increase the expression in order for PP to exert an effect at the genetic level that would adequately explain the liver weight and adiposity decrease, which was significant in both treatments. Hyperoside (quercetin 3-galactoside), quercetin 3-glucoside and quercetin 3-(6-malonylglucoside) were the main compounds present. Other authors [45] have shown that *C. olitorius* leaves also contain 5-caffeoylquinic acid (chlorogenic acid), 3,5-dicaffeoylquinic acid and quercetin 3-(6-malonylgalactoside).

### 2.5. Effect of PP Derived from Edible Crops

Rice (*Oryza sativa* L.) is one of the most important calorie sources for the majority of the human population and is normally consumed as white rice; other tonalities, such as black or red, are also available, and their pigmentation is due to anthocyanins [46]. Jang et al. [47] fed C57BL/6J mice a low fat diet (16.7% calories from fat), an HFD (45% of calories from fat) or an HFD enriched with 1% black rice extract for a seven-week period. Black rice extract contained cyanidin 3,5-diglucoside, cyanidin 3-glucoside and peonidin 3-glucoside as the major PP. Caloric intake and final BW were similar in all groups. Treatment decreased liver weight, serum TC, TAG and LDL, without affecting HDL or FFA (free fatty acids) relative to the HF group. HFD decreased hepatic PPARα mRNA expression, but increased with treatment, a tendency that was also apparent in CPT1A, ACOX and CYP4A10, all targets of PPARα. This pattern of gene expression favors microsomal ω-oxidation that is dependent on enzymes from the CYP450 family and mitochondrial β-oxidation, thereby inducing fatty acid catabolism and a preventive effect on hepatic steatosis. Others have shown that cyanidin is a PPAR agonist capable of directly binding to and activating all PPAR isoforms (with preference for PPARα), but that its major metabolites protocatechuic acid and phloroglucinaldehyde lose this ability [48].

Sorghum is an important crop cultivated around the world for human and animal consumption. Male C57BL/6 mice were fed either a regular or HFD (60% calories from fat); after eight weeks, HFD mice were further divided into three groups fed either 0.5% sorghum extract, 1% sorghum extract or saline (as an untreated HF group) for six weeks. Caloric intake, perirenal fat, serum TC, LDL, TAG and glucose were lower in the sorghum-treated groups. Both sorghum treatments increased adiponectin protein expression in adipose tissue, but only the 1% treatment increased PPARγ protein expression. The hypolipidemic and hypoglycemic effects of sorghum were due to an increase in PPARγ protein expression [49]. Although the authors do not report the exact composition of the administered extracts, others have described condensed tannins, phenolic acids (ferulic, syringic, protocatechuic, caffeic, *p*-coumaric and sinapic acid), anthocyanins (apigenin, luteolinidin, cyanidin and peonidin) and naringenin as the PP present in sorghum [50].

Flax (also called linseed, *Linum usitatissimum*) is cultivated to obtain flaxseeds or flaxseed oil; both are consumed by humans and animals and are regarded as an important source of essential fatty acids (α linoleic acid) [51]; lignans, a class of PP of which secoisolariciresinol is the most representative compound, are also present in flax and have shown important bioactivity. Fukumistu et al. [52] divided male C57BL/6 mice into four groups that were fed a low fat control diet (5% fat), an HFD (30% fat) or HFD enriched with 0.5% or 1.0% secoisolariciresinol diglucoside extracted from flaxseeds for a four-week period. Treatments reduced WAT mass as compared to the HFD group; liver weight was increased in all HFD groups without any effect from the treatments. Liver TAG and epididymal WAT mass increased in the HFD group; treatments prevented said increase. Liver cholesterol or glycemia did not change in any group. An increase in serum TC and TAG was also prevented by 1.0% treatment as compared to the HFD group. Hepatic mRNA expression of SREBP1c was decreased by both treatment doses; while that of ACOX, CPT1 and PPARα was unaffected. ACOX and PPARα mRNA expression in skeletal muscle was unchanged; a 1.0% dose increased CPT1 expression. In addition to the experiments performed in mice, the authors also demonstrated that enterodiol (which together with enterolactone is the main metabolite of secoisolariciresinol generated by bacteria in the colon of humans) increases PPARγ mRNA in 3T3-L1 adipocytes. Accordingly, these results suggest that flaxseed-extracted secoisolariciresinol induces a biological response related to SREBP1c inhibition, but its ability to modulate PPAR expression is dependent on an initial bacterial metabolism. *Peptostreptococcus* sp*.* and *Eubacterium* sp. have been identified as strains responsible for generating the aforementioned compounds in humans [53].

### 2.6. Effect of PP Derived from Medicinal Plants or Folk Remedies

Propolis or bee glue refers to a substance collected by bees from different plant sources that is used in the construction and fortification of beehives; humans have extensively used propolis for centuries because of its many medicinal properties and almost null toxicity [54]. Ichi et al. [55] studied mice fed an HFD (20% lard) with 0, 0.05 or 0.5% *w*/*w* propolis supplementation (control, low and high groups, respectively) for an eight-week period. WAT mass, hepatic and serum TC and TAG were decreased. The PPARγ protein level decreased in adipose tissue of the high group, while the hepatic PPARα protein level increased. Both treatments decreased hepatic SREBP1 protein; the high group also had decreased HMG CoA reductase protein. The authors proposed that the WAT decrease was due to PPARγ repression in adipocytes, since rosiglitazone (a PPARγ agonist) increases adipose mass. They also suggest that the hepatic SREBP1 protein decrease with the concomitant increase in PPARα protein favored catabolism over anabolism of TAG, thereby presenting a lower serum TAG concentration. Similarly, the serum TC decrease was due to downregulated HMG CoA reductase protein, since this enzyme is key to endogenous cholesterol synthesis and a pharmacologic target of the statin class of drugs aimed at lowering serum TC. Propolis is a complex mixture of flavonoids and phenolic acids (approximately half of the molecules); the rest is beeswax, volatiles and pollen [56]; the exact composition will depend on the specific characteristics of the collection site. Kumazawa et al. [57] analyzed (nuclear magnetic resonance) propolis collected from Japan, where the paper of Ichi et al. was performed, and report propolin A, B and E, prokinawan, nymphaeol A, B and C and isonymphaeol B, 3′-geranyl-naringenin as the prenylated flavonoids present in Japanese propolis. Due to the complexity of propolis, as well as non-PP molecules present, it is likely that the effects on PPAR described by Ichi et al. were not due to one molecule, but rather an intricate synergy between components that may vary according to propolis composition.

Licorice is extracted from the dried roots and rhizomes of different members of *Glycyrrhiza* species and has been used in Traditional Chinese Medicine for millennia; studies of its PP composition have revealed over 300 different compounds, of which flavanones and chalcones are major components [58]. Honda et al. [59] divided male Sprague-Dawley rats into two groups; the control group was fed an HFD; the treatment group was fed an HFD enriched with 2% licorice flavonoid oil for 21 d. Treatment decreased abdominal WAT, hepatic TAG content, plasma TAG and VLDL. Treatment increased hepatic PPARα mRNA expression, while suppressing SREBP1c expression, thereby pointing to an increase in fatty acid oxidation and a concomitant decrease in fatty acid synthesis. Although the authors report glabridin (an isoflavonoid) as the major flavonoid, the extract is likely composed of several other compounds.

Kuo et al. [60] administered an HFD to 40 Syrian golden hamsters for a seven-day period, after which the animals were further divided into five groups of eight animals each and fed for another seven d: Group A (control, vehicle only), Group B (250 mg/kg of ShanZha, *Crataegus pinnatifida*, dissolved in water), Group C (same as B + MK886 10 mg/kg day, a PPARα antagonist), Group D (200 mg/kg day clofibrate, a PPAR agonist as a positive control) and Group E (MK886 30 min before clofibrate as a negative control). TBW (total body weight), BAT percentage, TC, LDL and TAG decreased in the ShanZha and clofibrate groups; HDL increased in the ShanZha and clofibrate groups; epididymal WAT and food intake decreased in the ShanZha group; all respective to the control. PPARα protein expression in epididymal WAT increased in the ShanZha group; ShanZha combined with MK886 also increased PPARα expression relative to the control, but was less than the ShanZha group. Based on these findings, the authors suggest that the ShanZha effects were dependent on PPARα activation, since MK886 was able to partially inhibit the same effects induced by clofibrate. Considering that ShanZha extract contains ursolic acid (a triterpene) and seven PP, hyperoside (quercetin 3-galactoside), isoquercetin (quercetin 3-glucoside), epicatechin, chlorogenic acid, quercetin, rutin and protocatechuic acid [61], the effects reported were likely due to the combined effects of some or all of the aforementioned compounds.

Acacia trees are native to Australia and are rich in PP, particularly robinetinidol and fisetinidol. Ikarashi et al. [62] fed KKAy male mice a low fat diet (10% fat), an HFD (60% fat) or an HFD with the PP extract derived from *Acacia mearnsii* (2.5% or 5% *w*/*w*) for seven weeks. PP treatments decreased BW, glycemia, WAT mass, liver weight, liver TAG and liver cholesterol relative to the untreated HF group. The mRNA expression of PPARα was increased in skeletal muscle (5% group); both treatments also increased PPARδ expression. Hepatic PPARα mRNA expression increased in the 5% group; both treatments suppressed hepatic PPARγ expression. Both treatments upregulated PPARγ mRNA expression in WAT. The authors propose that acacia PP stimulate energy expenditure in muscle and liver, prevented liver lipid uptake and fatty liver and facilitate adiponectin synthesis. Altogether, this favors an anti-obesity and antidiabetic state.

*Selaginella tamariscina* Beauv. Spring is an herb used in traditional Oriental medicine with several positive effects described [63]. Zheng et al. [64] fed male Wistar rats a control or HFD (18% fat) for four weeks, after which the experimental animals were injected with streptozotocin in order to generate a type 2 diabetes model. Animals were then divided once more into five groups: a normal control group, a diabetic control group, a diabetic group treated daily with rosiglitazone (2 mg/kg BW, ig.) and three diabetic groups treated daily with total flavonoids of *S. tamariscina* (100, 200 or 400 mg/kg BW, ig.); treatments lasted an additional eight weeks. Untreated diabetic rats had increased serum TC, TAG, FFA and LDL; treatments with *S. tamariscina* PP prevented said increases and maintained similar values (or lower) to healthy control group. HDL decreased in untreated group; *S. tamariscina* PP increased HDL concentration. PPARγ protein expression in WAT was lower in the diabetic group; *S. tamariscina* PP (intermediate dose) increased its expression to values similar to the healthy control. PP exert a positive effect on PPAR expression in a diabetic model, which is another complex variable with profound metabolic derangement, such as macronutrient metabolism and hormonal balance; in humans, diabetes is a common occurrence related to HFD. Amentoflavone, 2,3-dihydroamentoflavone, hinokiflavone, neocryptomerin, podocarpusflavone, quercetin, apigenin and luteolin (the first five are biflavonoids) were reported in the treatments.

### 2.7. Effect of PP Administered as Pure Compounds

Caffeic acid and chlorogenic acid (3 *O*-caffeoylquinic acid) were administered in an HFD study where four groups of eight ICR mice were fed either a control diet, an HFD (37% calories from beef tallow), an HFD with 0.02% caffeic acid or an HFD with 0.02% chlorogenic acid. Both treatments decreased weight gain, visceral fat weight, plasma leptin, insulin, TAG and TC, while adiponectin increased. Hepatic PPARα mRNA expression increased (both treatments) and correlated with an increase in hepatic β-oxidation, which explains the visceral and plasma lipid decrease [65]. Although both compounds showed favorable results, chlorogenic acid was a more potent inductor of the measured responses and may indicate that the phenol moiety is more active as an aglycone. Results suggest it may be interesting to determine the effect of both compounds together to determine if they act in synergy.

Mangoes contain mangiferin, a PP of current interest that exerts positive health effects related to lipid metabolism. Guo et al. [66] fed four groups of hamsters either a control diet, an HFD (33% fat calories), an HFD and 50 or 150 mg/kg BW of mangiferin for an eight-week period. PP treatments decreased TBW, liver weight, visceral fat weight, serum TAG and FFA; caloric intake was higher in all groups compared to the control. HDL and LDL were higher in all groups (compared to the control). HF hepatic and muscular PPARα mRNA expression was lower than the control; mangiferin treatments prevented this downregulation. The PPARα mRNA decrease was related to serum lipid increase; the PPARα mRNA increase by mangiferin was related to the serum lipid decrease. Mangiferin is able to prevent or correct hypertriglyceridemia by modulating gene expression and favoring lipid catabolism by suppressing TAG-producing genes (SREBP1c, DGAT2 (diglyceride acyltransferase) and MTTP (microsomal triglyceride transfer protein)) and favoring lipid-catabolizing genes (PPARα, CPT1, LPL (lipoprotein lipase) and CD36). Since mangiferin is present in mangoes, it can be tempting to assume that whole mango consumption might be able to replicate the results seen here, since other compounds, such as fiber and carotenoids, are also present and can exert a synergistic effect on PPAR.

Ragab et al. [67] used an HF/high-sucrose diet in order to induce non-alcoholic fatty liver disease in male rats and to determine the effect of quercetin, *O*-coumaric acid and berberine (an alkaloid). Quercetin and its glycosides are found in different items, such as onions, apples and black tea [68]; *O*-coumaric acid is found in blueberries and black mulberries [69]; berberine is the main alkaloid found in *Berberis* species, from where it takes its name [70]. Fifty rats were divided into a control group (*n* = 10) and the HF/high-sucrose group (*n* = 40; 15% beef tallow, 10% sucrose and another 10% sucrose in the drinking water); after six weeks of feeding, the group was further divided into four groups of *n* = 10 and were fed for another six weeks: an untreated group, a quercetin group (50 mg/kg BW) and an *O*-coumaric acid (75 mg/kg BW) and berberine group (50 mg/kg BW); treatments were administered in 20% DMSO (quercetin and *O*-coumaric acid) or saline solution (berberine). The non-treated group had increased serum LDL, TC, TAG, hepatic total lipids, TC and TAG and decreased serum HDL. *O*-coumaric acid and berberine treatments decreased serum LDL, TC and TAG, while *O*-coumaric acid and berberine increased HDL, and quercetin decreased serum TC and TAG. All treatments decreased hepatic TC as compared to the HF/high-sucrose group. PPARγ mRNA expression was downregulated in all HF/high sucrose groups in adipose tissue; hepatic PPARγ mRNA expression was downregulated in the HF/high sucrose group, while *O*-coumaric acid and berberine prevented PPARγ downregulation. *O*-coumaric acid was better than quercetin at countering the negative effects of an HF/high sucrose diet. Similar results were found by Wein et al. [71]; rats were fed a control low fat diet (5% *w*/*w*) or an HFD (19% *w*/*w*) with or without 0.03% quercetin during a four-week period. PPARγ mRNA and transactivation was assayed in WAT, as well as adiponectin mRNA. Quercetin treatment increased adiponectin mRNA in WAT and serum concentration; however, PPARγ mRNA was decreased in WAT, suggesting that quercetin stimulates adiponectin through a PPARγ-independent pathway. Neither quercetin nor isorhamnetin (3-*O*-methylquercetin, quercetin’s major metabolite) are able to activate PPARγ.

Myricetin is a flavonoid available in different vegetable sources whose bioactive potential has been documented. Chang et al. [72] fed male Wistar rats a low fat control diet (11% calories from fat) or an HFD (45% calories from fat) for two weeks; after this period, myricetin was administered daily by oral gavage at 75, 150 or 300 mg/kg BW to the HFD group for eight additional weeks. Another group was administered 100 mg/kg BW of fenofibrate for the same period. Treatments decreased BW, WAT mass, plasma TC, plasma TAG, plasma FFA, liver TC and liver TAG (particularly at the 150 and 300 mg/kg BW doses), HDL was maintained similar to the control group. HFD decreased PPARα protein; myricetin treatment (at the highest dose) prevented this decrease. Expression of other proteins related to fatty acid oxidation (CYP4A and ACOX) showed a similar trend. Myricetin also prevented an increase in fatty acid (SREBP1) and cholesterol (SREBP2) synthesis-related proteins. Myricetin acted as an inductor of fatty acid catabolism that also blocks fatty acid and cholesterol anabolism.

Oleuropein is a phenolic secoiridoid glycoside found in olives and olive oil with several documented health benefits [73]. It was used to supplement (0.03%) HFD (40% energy from fat) fed to C57BL/6N mice for a ten-week period. HFD increased body weight gain and visceral adiposity; oleuropein treatment significantly hindered said increase. This was attributed to the downregulation of PPARγ mRNA expression in epididymal WAT, while also affecting galanin- and WNT10b-mediated signaling, which modulate adipogenesis and regulate PPARγ. Complementary experiments in 3T3-L1 preadipocytes showed that oleuropein prevented lipid accumulation in a dose-dependent manner, further confirming the results documented in mice [74].

Resveratrol is classified within the stilbene class of PP; it is found in important quantities in wine, and it is believed to be responsible for the protective effects of wine [75]. Andrade et al. [76] fed HFD (61% fat) to male FVB/N mice or an HFD + 30 mg/kg/d of resveratrol for a 60-day period. Resveratrol treatment suppressed TBF, TC, TAG and insulin secretion. HFD increased hepatic PPARγ mRNA expression; resveratrol maintained PPARγ mRNA expression similar to the control group (standard, low fat diet). mRNA expression of other genes related to adipogenesis (ACC and SREBP1c) also decreased, indicating that resveratrol exerted an antiadipogenic effect through hepatic gene modulation.

## 3. Conclusions

According to the information previously provided, it is apparent that most polyphenols from diverse vegetable sources are capable of inducing an increase in PPARα and γ mRNA or protein expression when administered as part of a high fat diet, particularly in liver and adipose tissue. Notable exceptions documented were catechins and quercetin. However, when assessing the effects of individual polyphenols, it must be taken into account that they are rarely ingested by themselves, but are part of a complex mixture where other classes of molecules, such as alkaloids, fiber, terpenes, phytosterols, vitamins, minerals and others, are also present and may synergize or antagonize them directly or through secondary signaling pathways. Other variables, such as endogenous or bacterial metabolism, will also impact on the quantified effect and, as with the particular case of flaxseed-derived polyphenols, may in fact be required. Further studies are merited to better document the intricate metabolic actions exerted by vegetable-derived polyphenols on human health in order to better direct them to our advantage.

## Figures and Tables

**Table 1:** Concise description of the studies described in the text highlighting the effects on PPAR and on body and serum lipids. References are listed by PP source.

Model	PPs Present	Effect on PPAR	Effect on Lipids	Ref.
***Effect of PP derived from fruit juices***				
C57BL6/J mice fed an HFD with *Moro* orange juice (12 weeks)	cyanidin 3-glucoside and cyanidin 3-(6″-malonylglucoside) * [18]	↑ PPARα mRNA in liver	↓ weight gain, TAG, TC and hepatic steatosis	[19]
Syrian golden hamsters fed an HFD with noni juice (*Morinda citrifolia* L., 6 weeks)	phenolic acids (gentisic, *p*-hydroxybenzoic, chlorogenic, caffeic, *p*-anisic, ferulic and gallic acid) and flavonoids (hesperidin, naringin and epicatechin)	↑ PPARα mRNA in liver	↓ TC, TAG, ↑ HDL, ↓ hepatic TC and TAG	[20]
***Effect of PP derived from other beverages***				
Wistar rats fed an HFD with green tea PP (26 weeks)	(−) epicatechin, (−) epicatechin-3-gallate, (−) epigallocatechin, (−) epigallocatechin-3-gallate, (+) catechin and (+) gallocatechin * [22]	↑ PPARγ mRNA and ↓ PPARγ phosphorylation in adipose tissue	↓ TC and TAG	[21]
C57BL/6 mice fed HFD with green tea-extracted catechins plus exercise (15 weeks)	epigallocatechin gallate, epigallocatechin, epicatechin gallate, epicatechin, gallocatechin, gallocatechin gallate and others	no effect on PPARα/δ as direct ligands	↓ body and visceral fat, serum TC and TAG	[23]
Sprague-Dawley rats fed HFD with green tea, black tea or epigallocatechin gallate (6 months)	epigallocatechin gallate	green and black tea ↑ hepatic PPARα mRNA; epigallocatechin had no effect; epigallocatechin ↑ PPARγ mRNA expression in adipose		[24]
C57BL/6 mice fed HFD with caffeine-free coffee PP (15 weeks, mRNA expression determined at the second week)	5-caffeoylquinic acid, 3,5-dicaffeoylquinic acid, 5-feruloylquinic acid and other isomers	no direct effect on any PPAR isoform in liver, WAT and BAT	↓ weight gain, WAT, liver weight and liver lipids	[25]
Swiss mice fed HFD with yerba maté (*Ilex paraguariensis*) aqueous extract (8 weeks)	5-caffeoylquinic acid and caffeic acid (caffeine and theobromine also present)	↑ PPARγ mRNA in WAT and BAT	↓ BW, TC, LDL and TAG	[27]
***Effect of PP derived from berries***				
Syrian golden hamsters fed HFD with blueberry pomace and blueberry pomace ethanolic extracts (3 weeks)	delphinidin, cyanidin, peonidin and malvidin * [32]	↑ PPARα mRNA in liver	↓ VLDL and TC	[31]
Human-derived HepG2 cells incubated with oleic acid and mulberry (*Morus indica* L.) water extract (24 h)	rutin, protocatechuic acid, epigallocatechin gallate, epicatechin, caffeic acid, hydroxyflavin, catechin, naringenin, quercetin, *p*-coumaric acid, resveratrol, hesperetin, gallic acid, ferulic acid and gossypin	↑ PPARα protein expression	↓ cholesterol and TAG synthesis	[34]
Wistar rats fed HFD with mulberry leaf extract (7 weeks)	quercetin and kaempferol	↑ PPARα/δ mRNA in liver	↓ TAG and FFA	[35]
***Effect of PP derived from other edible plants or fruits***				
C57BL/6J mice fed HFD with table grape PP	glycosides of delphinidin, cyanidin, petunidin, peonidin and malvidin	↓ PPARγ mRNA in liver	↓ liver weight and TAG	[36]
3T3-L1 adipocytes and C57BL/6J mice fed HFD with verbascoside or *Lippia citriodora* extract	verbascoside	↑ PPARα and PPARγ in 3T3-L1 adipocytes	↓ WAT, TC, TAG and liver lipids	[38]
Sprague-Dawley rats fed FHD with olive fruit pulp (*Olea europaea* L.) acetate extract (28 day)	hydroxytyrosol and others	↑ PPARα protein expression in liver	↓ TC and LDL	[40]
Syrian golden hamsters fed HFD with litchi (*Litchi chinensis* Sonn.) flower water extract (6 weeks)	flavonoids, condensed tannins, anthocyanins and proanthocyanidins	↑ PPARα mRNA in liver	↓ TC and TAG	[43]
LDLr^−^/^−^ mice fed an HFD with dried *Corchorus olitorius* leaf (8 weeks)	hyperoside, quercetin 3-glucoside and quercetin 3-(6-malonylglucoside)	↑ PPARα mRNA in liver	↓ BW gain, liver weight and liver TAG	[44]
***Effect of PP derived from edible crops***				
C57BL/6J mice fed HFD with black rice extracts (7 weeks)	cyanidin 3,5-diglucoside, cyanidin 3-glucoside and peonidin 3-glucoside	↑ PPARα mRNA in liver	↓ TC, TAG, LDL	[47]
C57BL/6 mice fed HFD with sorghum extracts (8 weeks)	tannins, phenolic acids, anthocyanins * [50]	↑ PPARγ protein expression in liver	↓ TC, TAG, LDL	[49]
C57BL/6 mice fed HFD with flaxseed-extracted secoisolariciresinol (4 weeks)	secoisolariciresinol diglucoside, enterodiol	no direct effect on liver or muscle PPARα mRNA. ↑ PPARγ mRNA in adipocytes by enterodiol	↓ liver TAG, WAT, TC and TAG	[52]
***Effect of PP derived from medicinal plants or folk remedies***				
Wistar rats fed an HFD with propolis (8 weeks)	propolin A, B and E, prokinawan, nymphaeol A, B and C, isonymphaeol B, 3’-geranyl-naringenin and others * [57]	↓ PPARγ protein in WAT and ↑ PPARα protein in liver	↓ WAT, hepatic and serum TC and TAG	[55]
Sprague-Dawley mice fed HFD with licorice flavonoid oil (21 days)	glabridin	↑ PPARα mRNA in liver	↓ WAT, hepatic TAG, plasma TAG and VLDL	[59]
Syrian Golden hamsters fed an HFD with ShanZha *(Crataegus pinnatifida)* extract (7 d)	hyperoside, isoquercetin, epicatechin, chlorogenic acid, quercetin, rutin and protocatechuic acid * [61]	↑ PPARα protein expression in epididymal WAT	↓ TBW, BAT, TC, LDL, TAG, ↑ HDL and ↓ WAT	[60]
KKAy mice fed HFD with *Acacia mearnsii* extract (7 weeks)	robinetinidol and fisetinidol	↑ PPARα/δ mRNA in liver and muscle. ↓ PPARγ mRNA in liver, ↑ in WAT.	↓ BW, WAT, liver weight, TAG and cholesterol	[62]
Wistar rats (induced type 2 diabetes) with *Selaginella tamariscina* flavonoids (8 weeks)	amentoflavone, 2,3-dihydroamentoflavone, hinokiflavone, neocryptomerin, podocarpusflavone, quercetin, apigenin and luteolin	↑ PPARγ protein in WAT	↓ TC, TAG, FFA and LDL, ↑ HDL	[64]
***Effect of PP administered as pure compounds***				
ICR mice fed an HFD with 0.02% PP (8 weeks)	caffeic acid and chlorogenic acid	↑ PPARα mRNA in liver	↓ weight, visceral fat, TAG and TC	[65]
Syrian golden hamsters fed an HFD with mangiferin (8 weeks)	mangiferin	↑ PPARα mRNA in liver and muscle	↓ TBW, liver weight, visceral fat, serum TAG and FFA	[66]
Wistar rats fed an HF/high sucrose diet with quercetin and *O*-coumaric acid (6 weeks)	quercetin and *O*-coumaric acid	No effect on PPARγ mRNA in adipose; *O*-coumaric acid prevented a decrease on hepatic PPARγ mRNA	↓ LDL, TC and TAG ↑ HDL	[67]
Wistar rats fed an HFD with quercetin (4 weeks)	quercetin	No effect on PPARγ mRNA or activation in WAT		[71]
Wistar rats fed an HFD with myricetin (8 weeks)	myricetin	↑ PPARα protein in liver	↓ BW, WAT, plasma TC, TAG and FFA, liver TC and TAG	[72]
C57BL/6N mice fed HFD with 0.03% oleuropein (10 weeks)	oleuropein	↓ PPARγ mRNA in epididymal WAT	↓ weight gain and visceral adiposity	[74]
FVB/N mice fed HFD with resveratrol (60 day)	resveratrol	↓ PPARγ mRNA in liver	↓ TBF, TC, TAG	[76]

Arrows indicate an increase (↑) or decrease (↓) in the respective variable. An asterisk (*) indicates that the PP content was taken from a different source to best approximate the PP composition of the treatments used by the authors of the original paper, since the specific composition is not always provided.

## Abbreviations

ACAT	acetyl-coenzyme A acetyltransferase
ACOX	acyl coenzyme A oxidase
BAT	brown adipose tissue
BW	body weight
CPT	carnitine palmitoyltransferase
CYP450	cytochrome P450
CYP7A1	cholesterol 7α hydroxylase
DGAT	diglyceride acyltransferase
DMSO	dimethyl sulfoxide
FAS	fatty acid synthase
FFA	free fatty acids
GPAT	glycerol-3-phosphate acyl transferase
HDL	high density lipoprotein
HF	high fat
HFD	high fat diet
HMG CoA	hydroxymethylglutaryl coenzyme A
LDL	low density lipoprotein
LPL	lipoprotein lipase
MTTP	microsomal triglyceride transfer protein
PP	polyphenols
SREBP	sterol regulatory element-binding protein
TBW	total body weight
TAG	triacylglycerols
TC	total cholesterol
UCP	uncoupling protein
VLDL	very low density lipoprotein
WAT	white adipose tissue
